# Predictive models based on machine learning for bone metastasis in patients with diagnosed colorectal cancer

**DOI:** 10.3389/fpubh.2022.984750

**Published:** 2022-09-20

**Authors:** Tianhao Li, Honghong Huang, Shuocun Zhang, Yongdan Zhang, Haoren Jing, Tianwei Sun, Xipeng Zhang, Liangfu Lu, Mingqing Zhang

**Affiliations:** ^1^Tianjin Union Medical Center, Tianjin Medical University, Tianjin, China; ^2^Academy of Medical Engineering and Translational Medicine, Tianjin University, Tianjin, China; ^3^Department of General Surgery, Tianjin Hongqiao Hospital, Tianjin, China; ^4^Department of Colorectal Surgery, Tianjin Union Medical Center, Tianjin, China; ^5^Tianjin Institute of Coloproctology, Tianjin, China; ^6^Department of Spinal Surgery, Tianjin Union Medical Center, Tianjin, China; ^7^The Institute of Translational Medicine, Tianjin Union Medical Center of Nankai University, Tianjin, China; ^8^Nankai University School of Medicine, Nankai University, Tianjin, China

**Keywords:** predictive model, artificial intelligence, colorectal cancer, machine learning, bone metastasis

## Abstract

**Background:**

This study aimed to develop an artificial intelligence predictive model for predicting the probability of developing BM in CRC patients.

**Methods:**

From SEER database, 50,566 CRC patients were identified between January 2015 and December 2019 without missing data. SVM and LR models were trained and tested on the dataset. Accuracy, area under the curve (AUC), and IDI were used to evaluate and compare the models.

**Results:**

For bone metastases in the entire cohort, SVM model with poly as kernel function presents the best performance, whose accuracy is 0.908, recall is 0.838, and AUC is 0.926, outperforming LR model. The top three most important factors affecting the model's prediction of BM include extraosseous metastases (EM), CEA, and size.

**Conclusion:**

Our study developed an SVM model with poly as kernel function for predicting BM in CRC patients. SVM model could improve personalized clinical decision-making, help rationalize the bone metastasis screening process, and reduce the burden on healthcare systems and patients.

## Introduction

Colorectal cancer (CRC) is a common malignant tumor, ranked the third most malignant tumor worldwide (1, 2). Distant metastasis is the leading cause of death in CRC patients (3), accounting for approximately 50% of patients after CRC surgery (4). The most common metastatic site of CRC is the liver or lung, while bone metastases are rare with an incidence of only 3–7% (5, 6). Patients with bone metastases have a poor prognosis, with a 5-year survival rate of < 5% and a median survival of 5–21 months (7–9).

Due to the low incidence and insignificant initial symptoms, bone metastases of CRC are difficult to diagnose at an early stage. On the one hand, compared with the low incidence of bone metastases in CRC patients, the incidence at autopsy is higher, reaching 10.7–23.7% (10). On the other hand, bone metastases are identified by further imaging or pathological examination after the occurrence of skeletal-related events (SREs) in CRC patients (11), but the median time to SREs is 2 months after the onset of bone metastases (7). Therefore, bone metastases may not be diagnosed on time in many CRC patients. Due to delayed diagnosis, patients may miss the optimal treatment time, leading to further disease progression and poor prognosis. Therefore, it is significant to predict the occurrence of bone metastasis in CRC patients.

Several predictive models for developing bone metastasis in CRC patients have been reported in previous studies (12–14). However, the performance of these models is hardly satisfactory because they are based on simple LR regression models, which may be unsuitable for predicting bone metastases. In addition, these models only identified independent risk factors associated with developing bone metastasis from CRC but did not assess the importance of each factor. Recently, artificial intelligence (AI) models based on machine learning (ML) algorithms have been increasingly used in clinical practice (15, 16). Among them, support vector machine (SVM) and other prediction models based on machine learning are better at predicting the distant metastasis of tumors, such as gastric cancer, thyroid cancer, and prostate cancer (17). SVM used in this study is a binary classification model whose basic model is a linear classifier defined by maximizing the interval on the feature space. SVM can be transformed into a non-linear classifier using the kernel method. SVM learning strategy is to maximize the interval, which can be translated into a convex quadratic programming problem and SVM learning algorithm is the optimization algorithm for solving the convex quadratic programming (18). Notably, SVM has some advantages for solving small sample high-dimensional problems. However, there are remain no studies using artificial intelligence models to predict bone metastasis in CRC patients.

Therefore, this study used population-based data to identify risk factors associated with bone metastasis in CRC patients and then build an artificial intelligence model to predict disease occurrence and help clinicians detect bone metastases in a timely manner. This can provide patients with personalized clinical strategies and promote rational allocation of healthcare resources.

## Materials and methods

### Study population

**Figure 1:**
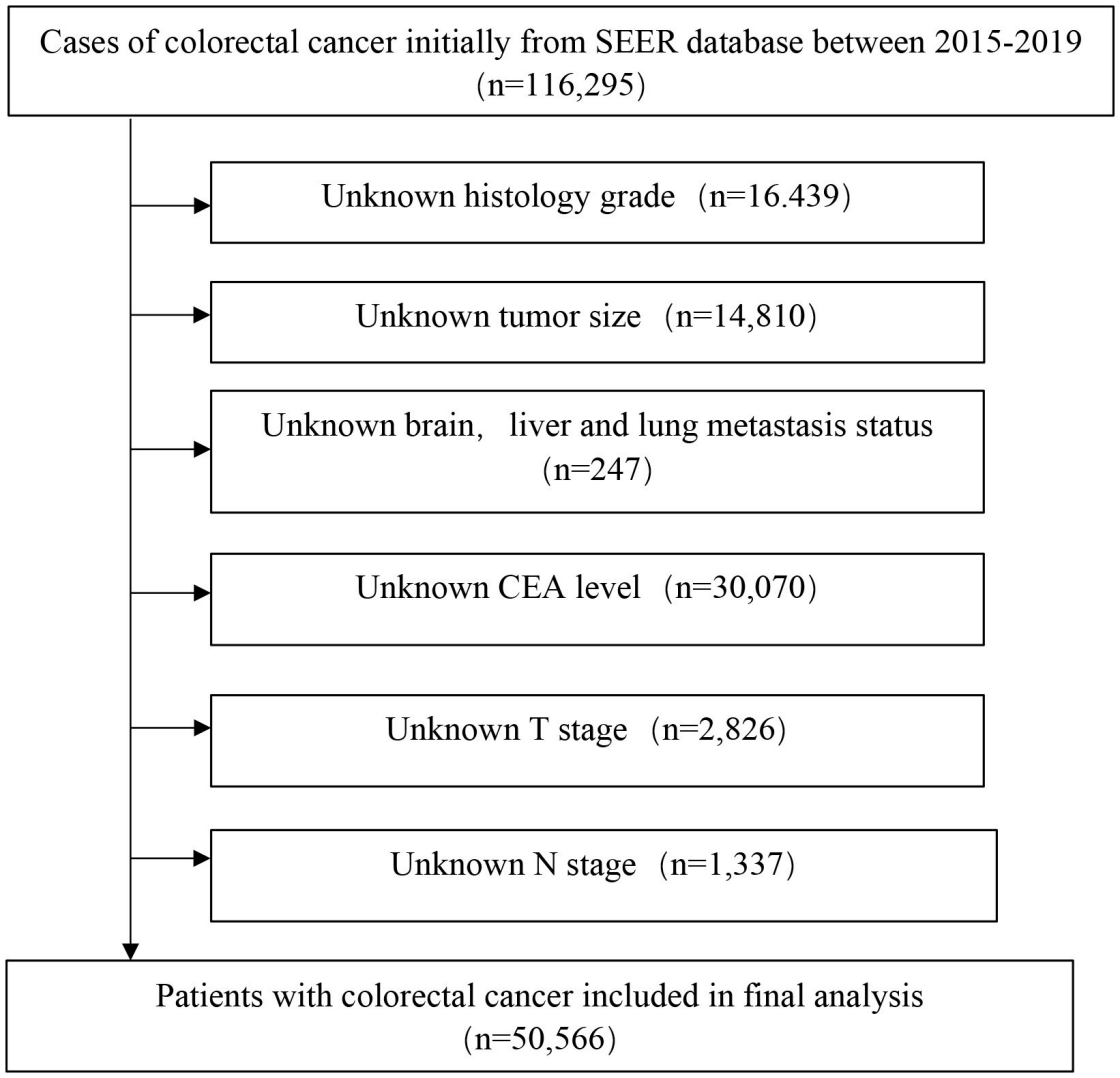
The analytical cohort and exclusion criteria.

This study was based on SEER database, and patient data were collected from “SEER Research Plus Data, 17 Registries, Nov 2021 Sub (2000–2019)” using SEER^*^stat 8.4.0 software and then extracted from the database between January 2015 and December 2019. SEER database covers 28% of the US population and includes information regarding cancer incidence, survival outcome, and treatment strategy from 17 population-based cancer registries. The patient selection procedure is displayed in Figure 1, and informed consent was not required as the patients were anonymized before publication. This study was approved by the Ethics Committee of Tianjin Union Medical Center.

The inclusion criteria were 1) primary CRC cases with histological confirmation, 2) histological classification: adenocarcinoma (icd-o-3:8140 to 8144, 8210 to 8213, 8220 to 8221, 8260 to 8263, 8551–8574) mucinous adenocarcinoma (MC, icd-o-3: 8480, 8481), seal ring cell carcinoma (SRCC, icd-o-3:8490), and 3) with a clear record of bone metastases. The exclusion criteria were (1) unknown information about the size, location, grade, The American Joint Committee on Cancer (AJCC) TNM stage(8th), T stage, N stage, surgery information, extraosseous metastasis, and bone metastatic status, and (2) CRC was not the first tumor.

### Data selection

All CRC patients were definitively diagnosed by pathologic examination, and BM was confirmed by imaging examination and/or pathologic examination. A total of 17 population, clinicopathological, serological indicator, extraosseous metastasis, and treatment variables were included. Population variables included age and sex, clinicopathology variables included site, size, grade, histology, AJCC TNM stage, T stage, N stage, and M stage, and serological indicators included carcinoembryonic antigen (CEA) levels. Extraosseous metastasis involves bone, brain, liver, and lung metastasis. All methods were conducted according to SEER database relevant guidelines.

### Model establishment

**Figure 2:**
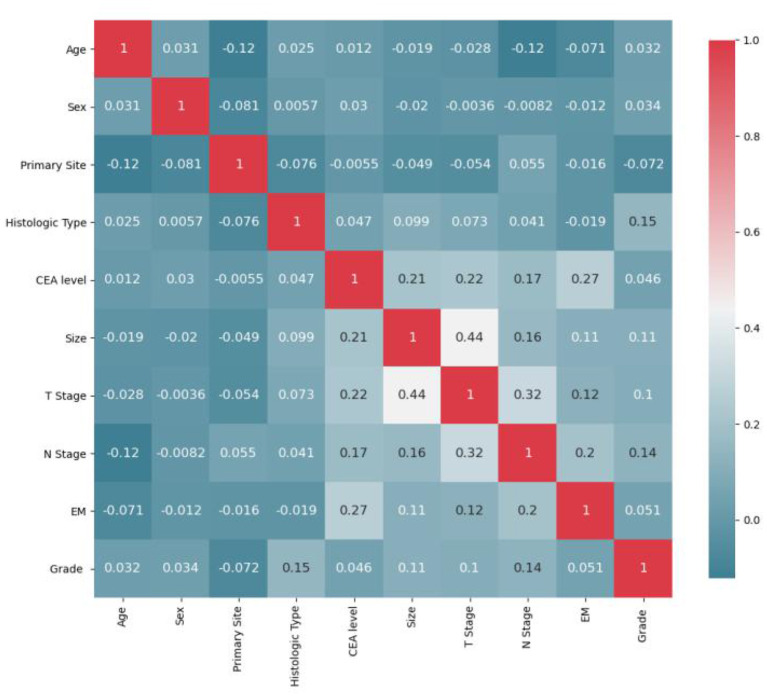
Feature correlation heatmap after initial preprocessing.

**Figure 3:**
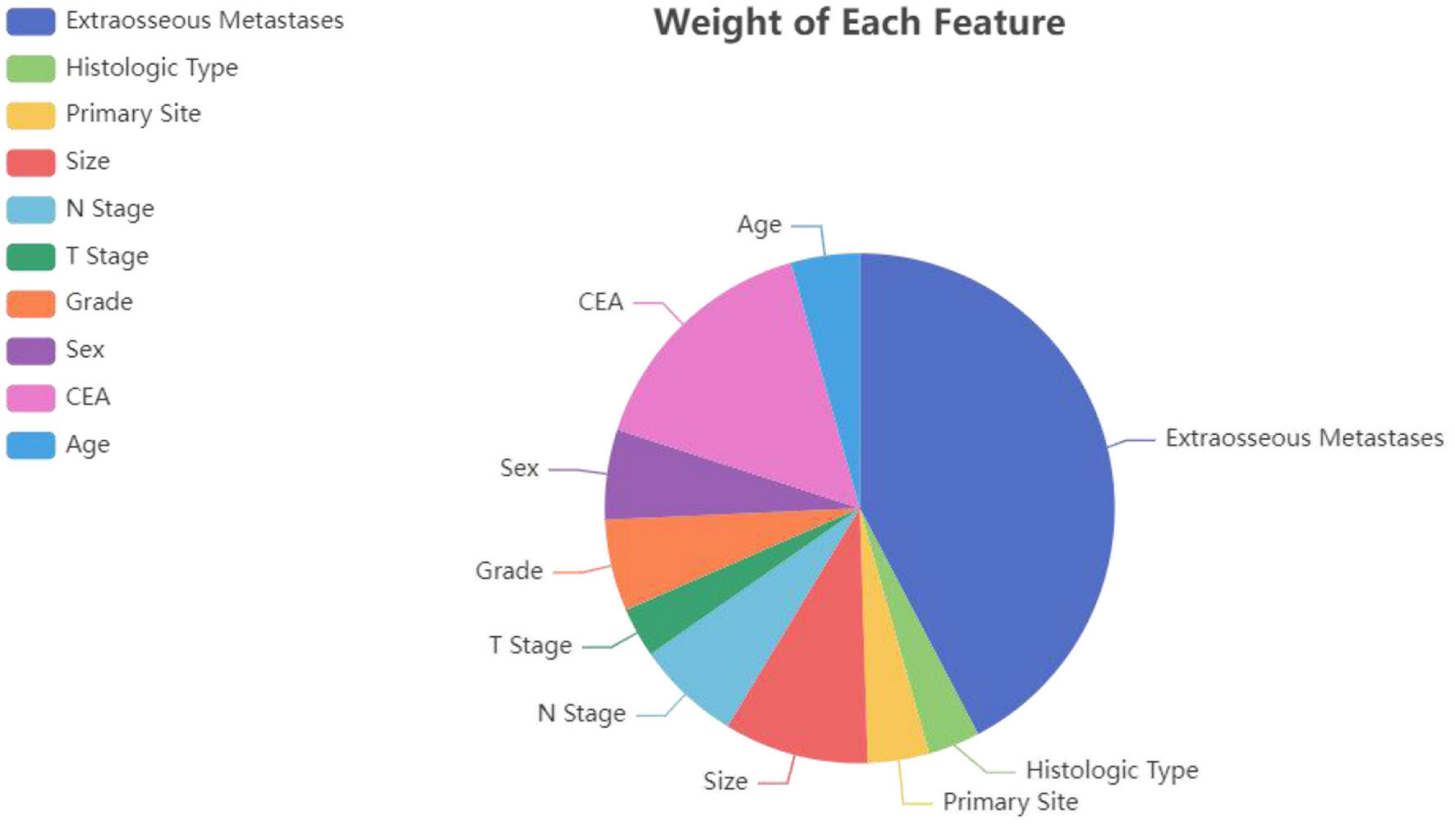
The influence weight of each factor calculated by the random forest algorithm.

All statistics were calculated using python (version 3.8). First, the initial data were preprocessed (12, 19). (1) Continuous variables: “Age” was divided into “>60 years” and “ <60 years”; “Size” was divided into “>2 cm,” “2–5 cm” and “>5 cm.” (2) Categorical variables: “Grade” was divided into “Grade I-II”, “Grade III-IV”; “T stage” was divided into “T1/2” and “T3/4”; “N stage” was divided into “N0” and “N1/2.” (3) Due to the small sample size and unbalanced distribution of the original distant metastasis variables (including lung metastasis, liver metastasis and brain metastasis) in SEER database, we added the variable of extraosseous metastasis for later model calculation. Pearson correlations between ten variables were calculated, and heatmaps were drawn. As Figure 2 displays, T stage strongly correlates with tumor size. For the features involved in the calculation to have low correlation, it is necessary to remove T stage or size, and the principle of feature removal is to remove the feature with less weight in the model calculation. The weight of each feature calculated by the random forest is presented in Figure 3. The figure shows that T stage occupies the smallest weight, implying that it is the is least important feature in the model analysis, so it is reasonable to remove T stage feature. To sum, nine features were included: age, sex, primary site, histologic type, CEA, size, N stage, extraosseous metastases (EM), and grade. Considering that the extreme imbalance of this sample (200:1) is likely to affect the model performance, it is necessary to adopt some sampling strategies. SMOTE Tomek was used in the training set as an Integrated Sampling method, and then the dataset was divided into a training set and a test set according to a ratio of 8:2.

**Figure 4:**
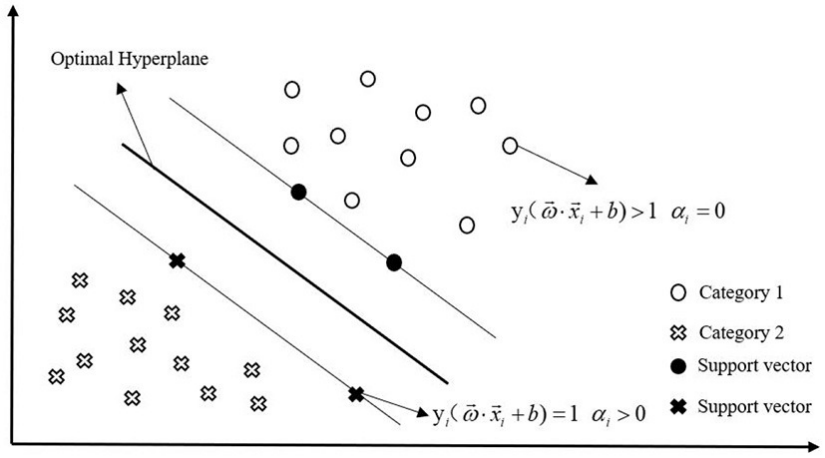
Schematic diagram of SVM.

SVM, LR, decision tree (DT), random forest (RF), and Extreme Gradient Boosting (XGB) models were used to analyze the data. To select a model with good results, we also include model comparison as part of the study. As a binary classification model, SVM aims to find the optimal hyperplane to partition the samples (Figure 4), the learning strategy is to maximize the interval, and the solution of the model must be transformed into a convex optimization problem. The basic principle is to map the sample training data from the low-dimensional space to the high-dimensional space. Consequently, the sample training data is linearly separable and then the boundaries are linearly partitioned. For the sample (*x*_*i*_, *y*_*i*_) and the hyperplane$\left(\vec{\omega },b\right)$, the geometric interval is defined as follows.


$$\begin{array}{l} \gamma_{i}=y_{i}\left(\frac{\vec{\omega }}{∥\vec{\omega }∥}\cdot {\vec{x}}_{i}+\frac{b}{∥\vec{\omega }∥}\right) \end{array}$$

Under the premise of correctly classifying the samples, when the geometric distance is the largest, the obtained separation hyperplane is optimal. The constraints are as follows:


$$\begin{array}{l} \underset{\omega ,b}{\max}\text{ Υ}\\ \;s.t.y_{i}\left(\frac{\vec{\omega }}{\left||\vec{\omega }|\right|}\cdot {\vec{x}}_{i}+\frac{b}{\left||\vec{\omega }|\right|}\right)\geq \text{Υ},i=1,2,\cdots ,N \end{array}$$

Using the decision boundary function, it can be transformed into the following:


$$\begin{array}{l} \;\underset{w,b}{\min}\frac{∥\vec{\omega }{∥}^{2}}{2}\\ \text{s}.\text{t}.y_{i}\left(\vec{\omega }\;\cdot \;{\vec{x}}_{i}+b\right)\geq 1,\;i=1,\;2,\ldots ,N \end{array}$$

After introducing the Lagrange operatorα_*i*_, it can be transformed into the following:


$$L\left(\vec{\omega },b,\alpha \right)=\frac{1}{2}∥\vec{\omega }{∥}^{2}-\overset{N}{\underset{i=1}{\sum }}\alpha_{i}\left(y_{i}\left(\vec{\omega }\cdot {\vec{x}}_{i}+b\right)-1\right)\left(\alpha_{i}\geq 0\right)$$

By taking the partial derivative of a function $L\left(\vec{\omega },b,\alpha \right)$ with respect to$\vec{\omega }$and*b*, we can obtain a function aboutα_*i*_.Let this function be 0 to find the optimal solution, and the optimal hyperplane formula can be obtained as follows.


$$\begin{array}{l} {\vec{\omega }}^{*}\cdot \vec{x}+b^{*}=0 \end{array}$$

Its corresponding Lagrange operator is optimal, denoted as$\alpha_{i}^{*}$. At this point the classification decision function is listed below.


$$f\left(\vec{x}\right)=sign\left({\vec{\omega }}^{*}\vec{X}+b^{*}\right)=sign\left(\overset{N}{\underset{i=1}{\sum }}\alpha_{i}^{*}y_{i}{\vec{x}}_{i}\cdot \vec{x}+b^{*}\right)$$

Using classification decision functions, samples can be classified, which is known as SVM. However, for non-linear problems, a kernel function is required. The function of kernel function is mainly to realize the mapping from a feature space in the support vector machine to another feature space and convert the inner product of high-dimensional vectors into the inner product of low-dimensional vectors.

In the model of LR, the goal of training is to find the best weight and bias for each feature so that the error is minimized. DT is a supervised machine learning algorithm based on if-then-else rules. In the model of RF, it is trained to obtain multiple decision trees by randomly putting back the samples sampled, and finally the results of each decision tree are summed using Bagging algorithm. The above model is built by python to learn the dataset's features.

### Model improvement

**Figure 5:**
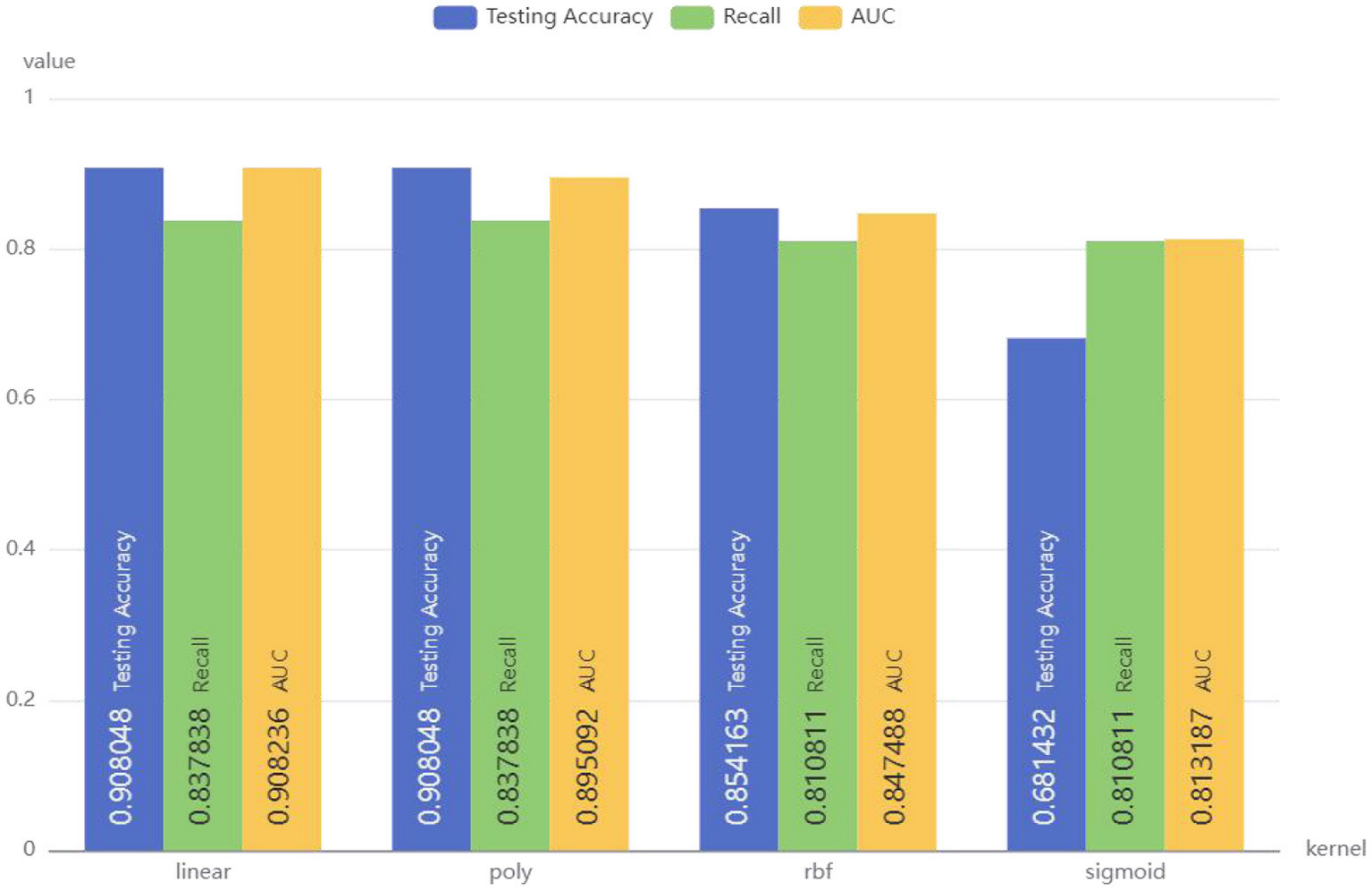
Performance of SVM models with different kernel functions.

Linear, poly, rbf, and sigmoid were used as kernel functions for SVM model, and the results are demonstrated in Figure 5. SVM model with poly as the kernel function showed the best performance, with an accuracy of 0.908048, recall of 0.837838, and AUC of 0.908236. Therefore, the linear kernel function was selected to build the SVM model to predict whether CRC patients have bone metastasis.

To achieve better model performance, a random search method was used for parameter optimization. After parameter optimization, SVM's Accuracy is 0.908, Recall is 0.838, and AUC is 0.926, demonstrating superior performance to previous models.

## Results

### Demographic and pathological characteristics

A total of 50,566 CRC patients were included in this study. At initial diagnosis, 50,325 patients (99.5%) had no bone metastases and 241 (0.5%) had bone metastases. All patients were randomized into a training set (*n* = 40,452) and a test set (*n* = 10,114) in a ratio of 8:2, and their clinical and pathological characteristics are listed in Table 1.

**Table 1:** Clinical and pathological characteristics of training and test sets.

**Variables**	**Training set**		**Test set**		
	**NBM(*n* = 40,248) %**	**BM(*n* = 204) %**	**NBM(*n* = 10,077) %**	**BM(*n* = 37) %**	***p*–value**
Age					0.714
<60	13847(34.4)	78(38.2)	3447(34.2)	15(40.5)	
>60	26401(65.6)	126(61.8)	6630(65.8)	222(59.5)	
Sex					0.182
Male	21857(54.3)	116(56.9)	5399(53.6)	20(54.1)	
Female	18391(45.7)	88(43.1)	4678(46.4)	17(45.9)	
Primary tumor site					0.922
Colon	30131(74.9)	135(66.2)	7552(74.9)	20(54.7)	
Rectal	10117(25.1)	69(33.8)	2525(25.1)	17(45.9)	
Size					0.91
<2 cm	4936(11.5)	1(0.5)	1150(11.4)	2(5.4)	
2–5 cm	21397(53.2)	108(52.9)	5385(53.4)	16(43.2)	
>5 cm	14215(35.3)	95(46.6)	3542(35.1)	19(51.4)	
Histology					0.947
Adenocarcinoma	37405(92.9)	189(92.6)	9366(92.9)	34(91.9)	
Mucosal adenocarcinoma	2542(6.3)	8(3.9)	633(6.3)	1(2.7)	
Signet-ring cell carcinoma	301(0.7)	7(3.4)	78(0.8)	2(5.4)	
T stage					0.839
T1/2	10411(25.9)	28(13.7)	2616(26)	4(10.8)	
T3/4	29837(74.1)	176(86.3)	7461(74)	33(89.2)	
N stage					0.108
N0	22046(54.8)	60(29.4)	5607(55.6)	10(27)	
N1/2	18201(45.2)	144(70.6)	4470(44.4)	27(73)	
Grade					0.566
Grade I–II	34486(85.7)	140(68.6)	8655(85.9)	25(67.6)	
Grade III–IV	5762(14.3)	64(31.4)	1422(14.1)	12(32.4)	
CEA level					0.242
Negative	23446(58.3)	32(15.7)	5930(58.8)	5(13.5)	
Positive	16802(41.7)	172(84.3)	4147(41.2)	32(86.5)	
Extraosseous metastases					0.012
No	36227(90)	64(31.4)	9153(90.8)	6(90.6)	
Yes	4021(10)	140(68.6)	924(9.2)	31(9.4)	
Brain metastasis					0.497
No	40219(99.9)	196(96.1)	10071(99.9)	36(97.3)	
Yes	29(0.1)	8(3.9)	6(0.1)	1(2.7)	
Liver metastasis					0.019
No	36655(91.1)	79(38.7)	9249(91.8)	11(29.7)	
Yes	3593(8.9)	125(61.3)	828(8.2)	26(70.3)	
Lung metastasis					0.704
No	39263(97.6)	138(67.6)	9838(97.6)	20(54.1)	
Yes	985(2.4)	66(32.4)	239(2.4)	17(45.9)	

### Model analysis and variable influence on prediction

Pearson correlations between all variables were calculated, and heatmaps were drawn, revealing no significant correlations between variables (Figure 5). For the multivariate LR model with an enter variable selection method, six characteristics were identified as independent risk factors (Table 2), including primary tumor site (*p* < 0.001), size (*p* = 0.042), histology (*p* = 0.018), grade (*p* < 0.001), CEA level (*p* < 0.001), EM (*p* < 0.001). According to RF results (Figure 4), the top three most important factors affecting model prediction of BM are EM, CEA, and size. Notably, the influence weight of EM accounts for 42.32%, which may provide some basis for diagnosing clinical auxiliary BM.

**Table 2:** Multivariable logistic regression model with enter variable selection.

**Variables**	**OR (95% CI)**	***P***
**Age**		
<60 years	Reference	
>60 years	0.155(0.862–1.548)	0.333
**Sex**		
Male	Reference	0.72
Female	0.949(0.715–1.261)	
**Primary tumor site**		<0.001
Colon	Reference	
Rectal	1.88(1.39–2.543)	
**Size**		
<2 cm	Reference	
2–5 cm	11.96(1.661–86.47)	0.014
>5 cm	10.868(1.504–78.531)	0.018
**Histology**		
Adenocarcinoma	Reference	
Mucosal adenocarcinoma	0.699(0.341–1.433)	0.328
Signet-ring cell carcinoma	3.035(1.316–6.998)	0.009
**N stage**		
N0	Reference	
N1	1.123(0.815–1.548)	0.479
**Grade**		<0.001
Grade I–II	Reference	
Grade III–IV	2.118(1.537–2.92)	
**CEA level**		<0.001
Negative	Reference	
Positive	2.879(1.908–4.344)	
**Extraosseous metastases**		<0.001
No	Reference	
Yes	12.207(8.805–16.923)	

### Model performance

**Figure 6:**
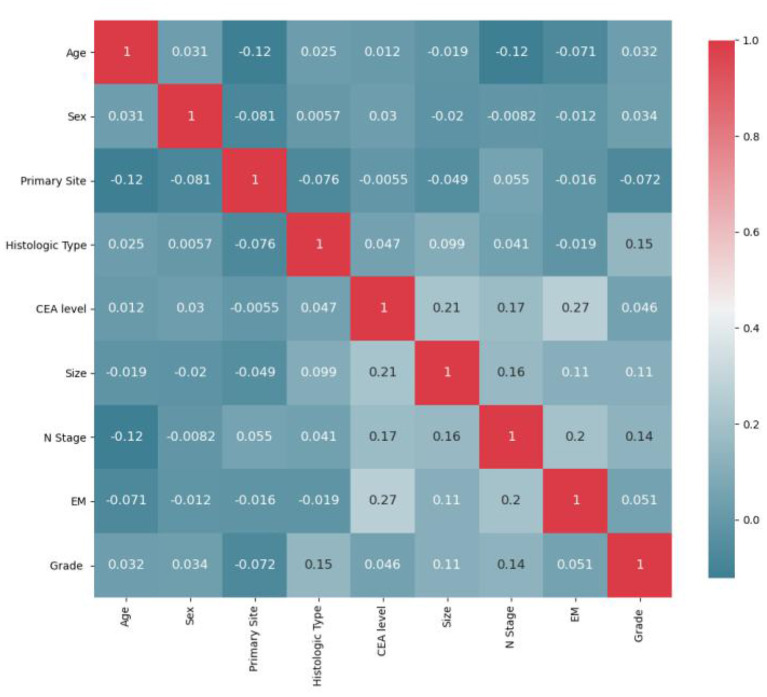
Results of Pearson correlation analysis between all variables. The heatmap shows the correlation between the variables.

**Figure 7:**
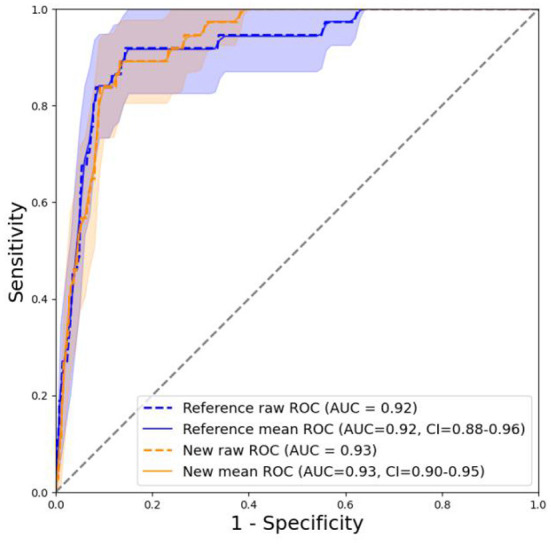
ROC curve, in which the new model refers to SVM and the old one refers to LR.

**Figure 8:**
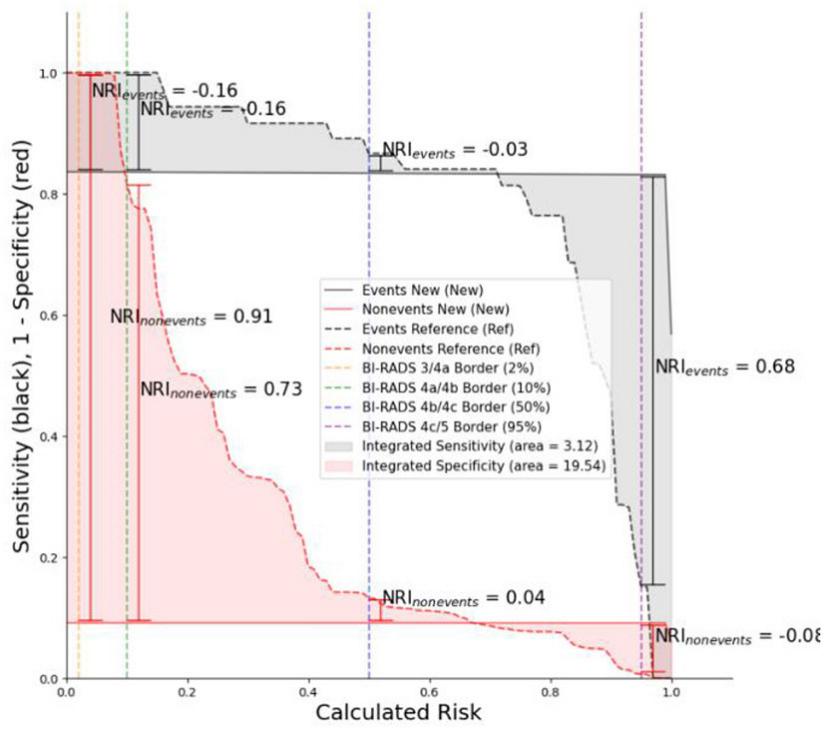
IDI curve, in which the new model refers to SVM and the old one refers to LR.

The training set was used to train the model, and the test set was used to test the accuracy and generalization ability of the model. The performance indicators of the evaluation model were AUC, Accuracy, and Recall. After comparing the performance of different kernel functions in the SVM model (Figure 6), the linear kernel function was selected. The results were compared and analyzed using SVM, LR, DT, RF, and XGB models. The performance comparison of different models is provided in Table 3, showing that SVM model is better than the other models and may be used clinically. Previous models have mostly used LR, and to better compare the improvements brought about by SVM model, ROC curves were plotted, and Integrated Discrimination Improvement (IDI) was calculated. As displayed in Figure 7, LR AUC is 0.92, and SVM AUC is 0.93, with an IDI of 22.66% (Figure 8), confirming that SVM model outperforms LR in this scenario.

**Table 3:** Comparing the prediction performances of different models for BM.

**Models**	**AUC**	**Accuracy**	**Recall**
SVM	0.926	0.908	0.838
LR	0.918	0.865	0.865
DT	0.770	0.850	0.703
RF	0.770	0.850	0.676
XGB	0.873	0.882	0.838

## Discussion

The incidence of bone metastasis in CRC patients is only 3–7% (6), but these patients have a poor clinical prognosis and often suffer from SREs, such as pathological fractures, severe bone pain, spinal cord compression, and hypercalcemia (20) which can seriously impair their function and quality of life, and even further affect the outcomes. Therefore, early identification and clinical intervention of bone metastasis are critical to prevent SREs and improve the clinical prognosis.

There remains a lack of accurate and effective methods to predict bone metastasis in CRC patients. Pathological diagnosis is the gold standard, but if the pathological diagnosis is unclear, the identification of bone metastasis in CRC patients relies on SREs and imaging examinations such as X-ray, CT, MRI, emission computed tomography (ECT), and positron emission tomography/computed tomography (PET/CT) (21, 22). However, these imaging modalities are expensive and associated with radiation risks, so they are not recommended as routine screening for CRC patients until SREs occur (12). For this reason, we developed an artificial intelligence model based on SVM algorithm to predict bone metastasis in CRC patients.

The advantage of this model is that it can effectively deal with the imbalance of medical data, as SVM algorithm can effectively solve the problem of inaccurate judgment results caused by small sample data in machine learning, which has stronger practicability (18, 23). In this study, this model displayed better accuracy and generalization than other models (LR, DT, and RF) and can be used to predict the occurrence of bone metastasis in CRC patients, which is helpful for doctors to make timely and effective clinical decisions.

Previous studies have reported risk factors associated with bone metastasis in CRC. Zheng et al. (21) conducted a retrospective study of 106 patients with bone metastasis of CRC, indicating that primary tumor location, lung metastasis, and serum CEA are independent risk factors. Moreover, compared with colon cancer and liver metastasis, colorectal cancer and lung metastasis were more likely to predict disease progression to bone metastasis. Wang et al. (13) determined that the degree of tumor differentiation, N stage, serum alkaline phosphatase (ALP), lactate dehydrogenase (LDH), CEA, liver and lung metastasis were risk factors for bone metastasis of CRC, and further developed a nomogram to evaluate the risk of bone metastasis in CRC patients. In addition, studies have shown that the most common risk factors for BM in CRC patients include cancer site, lymph node invasion, and lung metastasis (6).

In this study, EM, followed by CEA level and tumor size, were the top three most important factors for developing bone metastasis in CRC patients. Notably, EM has an influence weight of 42.32%, an important predictor of bone metastasis in CRC patients. Studies have depicted that about 25% of CRC patients have distant metastases at the time of diagnosis (24). In our study, EM occurred in 10% of CRC patients and was an independent predictor of bone metastasis in CRC patients, consistent with previous findings (12). Due to the low incidence and insidious symptoms of bone metastasis, it is often identified after the occurrence of SREs, when the disease has already advanced, so the treatment effect and prognosis are poor (25). In addition, due to the environment of a specific organ and its effect on tumor cell adhesion, CRC tends to metastasize first to the liver or lungs before the bones (8, 9, 26). Therefore, for CRC patients with extraosseous metastasis, regular health monitoring and follow-up may be helpful for the early identification of bone metastases.

Serum CEA is considered a specific biomarker for CRC, and its concentration is significantly elevated in patients with metastatic colon cancer (27–29). In this study, CEA level was an independent predictor of bone metastasis in CRC patients, consistent with previous findings (21). Higher CEA levels may be associated with distant metastasis of CRC and nerve infiltration (30). In addition, in the current AJCC TNM staging of CRC, T staging is determined by the depth of the tumor invading the intestinal wall rather than the tumor size, but previous studies have shown that solid tumors, including those of the gastrointestinal tract, exhibit the potential to spread not only during the vertical invasion but also during horizontal growth; (31). As the tumor size increases, the potential for metastasis is higher (32). A retrospective study by Luo et al. showed that tumor size was positively correlated with distant metastasis of rectal cancer (33). Similarly, our study depicted that size was an independent risk factor for bone metastasis of CRC, with a significantly higher incidence of bone metastases in tumors larger than 2 cm. This may provide some basis for diagnosing CRC patients with bone metastases in the clinic.

Nonetheless, this study has some limitations. First, since the model was not externally validated and was based on retrospective data, prospective cohort studies are needed to validate its accuracy and stability. Second, the model is based on an SVM algorithm, so it may be clinically difficult to interpret key features screened out by the model. In addition, since all study subjects were representative of the US population, the application of this risk model to other countries and ethnicities is limited.

Nowadays, with the rapid development of artificial intelligence technology, deep learning is widely applied in the detection and treatment of various diseases, such as cancer, diabetes, Alzheimer's disease and Parkinson's disease, and better results have been obtained (34, 35). In future research, it is planned to apply deep learning techniques in the prediction of bone metastasis occurring in colorectal cancer.

## Conclusion

This study developed and validated an artificial intelligence model based on machine learning algorithms to individually predict the occurrence of bone metastasis in CRC patients by using clinical characteristics and quantifying the major factors leading to the increased risk of bone metastases. Among them, EM, followed by CEA level and size, were the top three most important factors for bone metastasis in CRC patients. Compared with the traditional LR model, the prediction performance of SVM algorithm is better (IDI: 22.66%); consequently, it could be used to timely detect bone metastases providing patients with personalized treatment and allocating health resources more effectively.

## Data availability statement

The raw data supporting the conclusions of this article will be made available by the authors, without undue reservation.

## Ethics statement

The studies involving human participants were reviewed and approved by the Ethics Committee of Tianjin Union Medical Center. Written informed consent for participation was not required for this study in accordance with the national legislation and the institutional requirements.

## Author contributions

XZ, MZ, and LL conceived the idea for the study. LL and MZ were involved in planning and supervised data collection. HJ and SZ performed data collection. SZ, YZ, and HJ conducted data analysis. TL and HH drafted the manuscript. MZ, LL, and TS contributed to writing of manuscript. All authors have discussed and decided that this manuscript is the final version and agreed to publish it.

## Funding

This study was funded by Foundation of Tianjin Union Medical Center (grant number: 2016RMNK002 and 2019ZDXK01). This work was funded by Tianjin Key Medical Discipline (Specialty) Construction Project.

## Conflict of interest

The authors declare that the research was conducted in the absence of any commercial or financial relationships that could be construed as a potential conflict of interest.

## Publisher's note

All claims expressed in this article are solely those of the authors and do not necessarily represent those of their affiliated organizations, or those of the publisher, the editors and the reviewers. Any product that may be evaluated in this article, or claim that may be made by its manufacturer, is not guaranteed or endorsed by the publisher.
